# Emerging therapeutic benefit of platelet-rich fibrin as novel platelet concentrates in tissue engineering

**DOI:** 10.21542/gcsp.2024.46

**Published:** 2024-11-01

**Authors:** I Gde Rurus Suryawan, Andrianto, Arisya Agita, Anudya Kartika Ratri, Ricardo Adrian Nugraha

**Affiliations:** 1 Division of Interventional Cardiology, Department of Cardiology and Vascular Medicine, Faculty of Medicine Universitas Airlangga - Dr. Soetomo Academic General Hospital; 2 Division of Intensive and Acute Cardiovascular Care, Department of Cardiology and Vascular Medicine, Faculty of Medicine Universitas Airlangga. - Dr. Soetomo Academic General Hospital; 3 Department of Cardiology, Mitra Keluarga Kenjeran, Surabaya; 4 Division of Pediatric Cardiology, Department of Cardiology and Vascular Medicine, Faculty of Medicine Universitas Airlangga - Dr. Soetomo Academic General Hospital; 5 Department of Cardiology and Vascular Medicine, Faculty of Medicine Universitas Airlangga - Dr. Soetomo Academic General Hospital

## Abstract

**Background:** Treating irreversible cardiomyocyte loss following myocardial infarction presents several therapeutic challenges. While cell therapy shows promise as a regenerative treatment for infarcted cardiac tissue, different cell sources vary in their therapeutic potential. Adipose-derived stem cells (ADSCs) have emerged as an attractive option due to their accessibility, but their limited differentiation capacity remains a significant constraint. Recent evidence suggests that injectable platelet-rich fibrin may enhance this process by stimulating the differentiation of ADSCs into cardiomyocyte-like cells.

**Objective:** Analyse the benefit of injectable platelet-rich fibrin to accelerate the differentiation of adipose-derived mesenchymal stem cells into cardiomyocyte-like cells.

**Methods:** This study is a true experimental randomized pos *t*-test design study. Adipose-derived mesenchymal stem cells were isolated from adipose tissue and expanded in culture through four passages. The characteristics of adipose-derived mesenchymal stem cells were measured by the expression of CD 34-, CD 45-, and CD 105+ using flowcytometry. The samples were divided into 3 groups, i.e., negative control (*α*-MEM), positive control (differentiation medium) and treatment group (platelet-rich fibrin). The assessment of GATA-4 marker expression was conducted using flowcytometry on the fifth day and troponin was conducted using immunocytochemistry on the tenth day to determine the differentiation to cardiomyocyte. Statistical analysis was performed using Student’s t-tests and one-way ANOVA for data that demonstrated normal distribution as verified by the Shapiro–Wilk test.

**Results:** Flowcytometry on GATA-4 expression revealed significant difference on addition of platelet-rich fibrin compared with negative and positive controls (68.20 ± 6.82 *vs* 58.15 ± 1.23; *p* < 0.05; 68.20 ± 6.82 *vs* 52.96 ± 2.02; *p* < 0.05). This was supported by the results of immunocytochemistry on troponin expression which revealed significant difference between platelet-rich fibrin group compared with negative and positive controls (50.66 ± 7.2 *vs* 10.73 ± 2.39; *p* < 0.05; 50.66 ± 7.2 *vs* 26.00 ± 0.4; *p* < 0.05).

**Conclusion:** Injectable platelet-rich fibrin accelerates differentiation of adipose-derived mesenchymal stem cells into cardiomyocyte-like cells.

## Introduction

Coronary heart disease significantly impacts both life expectancy and quality of life. Cardiomyocytes in adults have a limited capacity to regenerate after coronary heart disease. This permanent cardiomyocyte damage results in impaired contractile function and increased fibroblast proliferation, leading to progressive non-ischemic ventricular remodeling. This remodelling process causes progressive ventricular dilatation can envetually lead to heart failure. Currently, there are no approved clinical therapies to regenerate damaged myocardium in coronary heart disease. Cell therapy thus represents a promising therapeutic approach for myocardial regeneration^1^.

Bone marrow-derived mesenchymal stem cells (BMSCs) were the first MSCs to be identified and remain the most extensively studied in the context of cardiovascular disease. Unfortunately, the use of BMSCs is invasive, painful, has a high morbidity and low success rate. In contrast, adipose-derived mesenchymal stem cells (ADSCs) can be isolated through less invasive procedures and demonstrate more favorable outcomes. ADSCs yield higher cell densities than BMSCs (5% *vs* 0.1% of nucleated cells)^2^.

Growth factors are crucial for maintaining stem cell self-renewal capacity, while blood-derived growth factors and nutrients promote stem cell proliferation. Platelets are the main source of growth factors that play a role in tissue regeneration. Platelet-rich fibrin (PRF) represents an advancement in platelet-based therapy. Unlike other platelet concentrates, PRF formation requires only centrifugation of whole blood without additives or gelifying agents^3^.

PRF contains a variety of growth factors. These growth factors trigger the movement, proliferation and differentiation of stem cells, such as in neovascularization and collagen synthesis. In addition, PRF also triggers soft tissue growth to accelerate healing and improve quality wound healing. Transforming growth factor-*β*1 (TGF-*β*1) triggers fibrosis while platelet-derived growth factor (PDGF) contributes to mesenchymal cell migration and survival. Insulin-like growth factor-1 (IGF-1) prevents apoptosis, Vascular endothelial growth factor (VEGF) stimulates vasculogenesis and angiogenesis, while epidermal growth factor (EGF) promotes cell proliferation and differentiation^4^.

AMSCs express markers of MSCs, namely CD10, CD13, CD29, CD34, CD44, CD54, CD71, CD90, CD105, CD106, and CD117. AMSCs do not express hematopoietic cell lineage markers such as CD45, CD14, CD16, CD56, CD61, CD62E, CD104, and CD106; and also endothelial cell lineages namely CD31, CD144, and von Willebrand factor. Morphologically, these cells are like fibroblasts and retain their shape after expansion in vitro. The similarity between AMSCs and BMSCs suggests that AMSCs originate from circulating BMSCs that infiltrate into the adipose compartment through the vessel wall^5^.

## Objective

While previous studies have demonstrated the role of platelets and their growth factors in cell differentiation, the effect of PRF on ADSC differentiation remains unexplored. This study investigates whether PRF supplementation influences ADSC proliferation and examines the capacity of injectable PRF to enhance ADSC differentiation into cardiomyocyte-like cells, as assessed by GATA4 and troponin expression levels.

## Methodology

### Ethical approval

This research had ethical approval from Institutional Review Board of Dr. Soetomo Academic General Hospital – Faculty of Medicine, Airlangga University (Institution Review Board number: IORG0007195; Reference Number: 1733/KEPK/XII/2019) issued on December 27th, 2019.

### Study design

This is an experimental *in vitro* study with the administration of plasma rich fibrin (PRF) in AMSCs culture with the aim of determining the effect of the addition of PRF on the ability of AMSCs to differentiate into cardiomyocyte-like cells. This type of research is true experimental with randomized randomization with a “pos *t*-test only control group design” approach.

### Study setting

The laboratory research was conducted at the Stem Cell Laboratory, Centre for Biomaterials and Tissue Bank, Dr. Soetomo Academic General Hospital between 1 January 2020–31 December 2021.

### Sample size

The experimental design adhered to three fundamental principles in biological sample processing: randomization, replication, and control. In this study, these three principles were applied as follows. Randomization was carried out before the research sample was divided into three groups. Replication was carried out after the research sample was divided into three groups. The principle of replication is to increase the accuracy of research results with minimal sample variation. Where, when applied to the principle of statistical data processing, replication must be carried out at least three times, this is done so that the calculation of the standard deviation can be carried out. This is in accordance with the principle of Triplicate calculation. The control arm included both negative and positive controls.

### Materials

1. Adipose-derived mesenchymal stem cells (AMSCs), namely mesenchymal stem cells (MSCs) obtained from minimal surgery procedures.
2. Bio Safety Cabinet class II
3. Centrifuge
4. Inverted Microscope
5. CO_2_ Incubator
6. Pipette aid and micropipette
7. Water bath
8. Hot plate magnetic stirrer
9. Dissecting set
10. Vacuum pump
11. 500 mL Beaker glass
12. *α*-MEM medium
13. Phosphate buffer saline (PBS) with fetal bovine serum (FBS)
14. Triple express
15. Collagenase
16. NaHCO_3_
17. Trypan Blue
18. 70% Alcohol
19. 15 mL and 50 mL conical tube
20. 5 cm and 10 cm petri dish
21. 24 wells and 12 wells of microplate
22. Unit filter 0,22 µm (Millipore)
23. CD34, CD45, CD105 staining
24. GATA-4 antibody
25. cTnT antibody
26. Cardiomyocyte differentiated medium, consisting of RPMI 1640, rice-derived recombinant human albumin, and L-ascorbic acid 2-phosphate
27. Platelet Rich Fibrin

### Experimental procedures

This research was conducted through 5 stages:

1. Isolation and culture of AMSCs from adipose tissue obtained from minimal surgery procedures.
2. Identification of AMSCs from adipose tissue by flow cytometry by observing the expression of CD 105 and the non-expression of CD 34 and CD 45.
3. Culture of AMSCs on MEM alpha medium and differentiation of cardiomyocytes.
4. Preparation and administration of PRF in *in vitro* cultured AMSCs in treatment group. Around 5 mL of whole venous blood is collected in each of the two sterile vacutainer tubes of 6 mL capacity without anticoagulant. The vacutainer tubes are then placed in a centrifugal machine at 3000 rpm for 10 min, after which it settles into the following layers: red lower fraction containing red blood cells, upper straw coloured cellular plasma and the middle fraction containing the fibrin clot. The upper straw coloured layer is then removed and middle fraction is collected, two mm below lower dividing line, which is the PRF. The mechanism which is followed here is that, fibrinogen which is initially concentrated in the high part of the tube, combines with the circulating thrombin due to centrifugation, to form fibrin. A fibrin clot forms in the middle of the tube, positioned between the sedimented red blood cells at the bottom and the acellular plasma at the top. Within this fibrin network, platelets are extensively entrapped.
5. Evaluation of tissue culture of AMSCs in the three groups with flowcytometry for GATA-4 and Immunocytochemistry for cTnT.

### Statistical analysis

Given the sample size was less than 30, data normality was assessed using the Shapiro–Wilk test. Statistical analysis employed either one-way ANOVA for normally distributed data or the Kruskal-Wallis test for non-normally distributed data. Post hoc comparisons between groups were performed using either Student’s *t*-test for normally distributed data or the Mann–Whitney U test for non-normally distributed data. Statistical significance was set at *p* = 0.05.

## Results

### Isolation and culture of AMSCs

**Figure 1.**
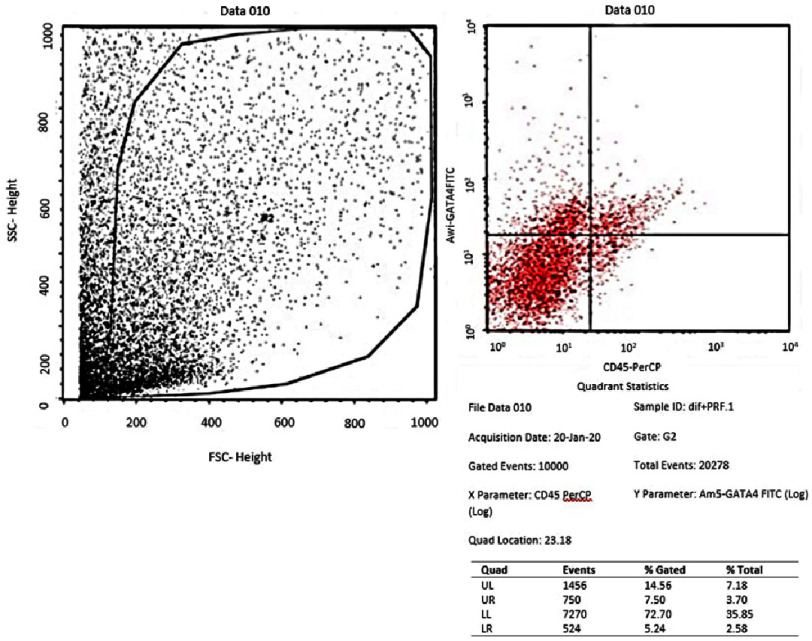
Flowcytometry of GATA-4 in the negative control group (*α*-MEM).

AMSCs were extracted from adipose tissue using a minimally invasive surgical method. This isolation procedure is adjusted to the standard stem cell laboratory at the Stem Cell Laboratory, Centre for Biomaterials and Tissue Bank, Dr. Soetomo Academic General Hospital. AMSCs were cultured and expanded through passage 4 to preserve their stemness properties while preventing spontaneous differentiation. The majority of cells were seen to have a spindle-like shape. Figure 1 shows a microscopic image of AMSCs culture starting from the time of isolation (passage 0) to the final culture (passage 4).

### Phenotype Characterization of AMSCs

**Figure 2.**
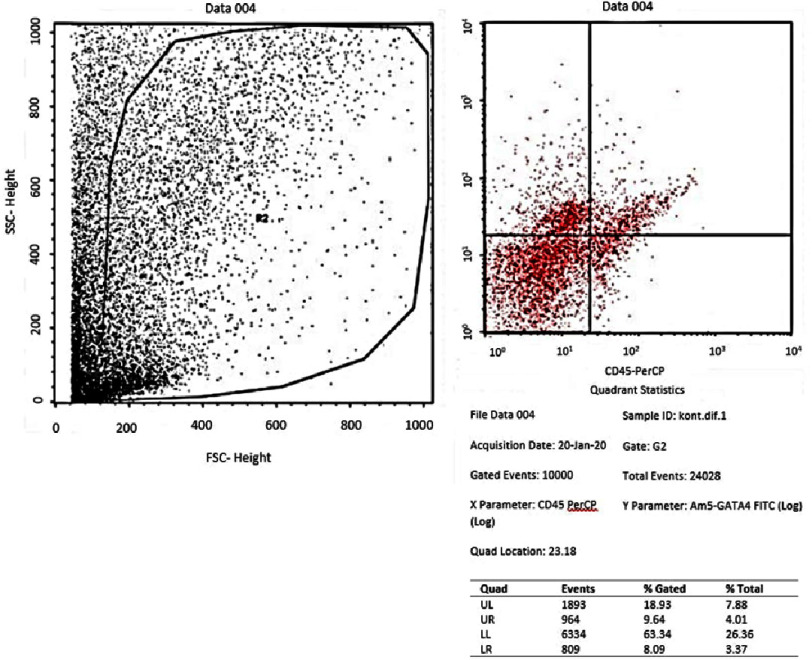
Flowcytometry of GATA-4 in the positive control group (medium of differentiation).

Based on the minimum criteria for the characterization of MSCs by The International Society for Cellular Therapy (ISCT), the majority of cells showed positive expression of CD73, CD90, and CD105, and negative expression of CD11_b_ or CD14, CD19, CD34, CD45, and HLA-DR. In this study, AMSCs that had been taken from adipose tissue were examined for characterization of MSCs in the fourth passage culture. AMSCs culture showed positive expression of CD105+ and negative expression of CD34- and CD45- by flow cytometry examination. Phenotype identification is based on cell morphology viewed by an inverted microscope (x100 magnification) (Figure 2).

### Flow cytometric evaluation of GATA-4 during cardiomyocyte differentiation

GATA-4 examination at the cardiomyocyte-like cells differentiation stage was carried out on day 5 because AMSCs already appeared confluent. To determine the difference test between the three groups, we began with a distribution test to determine the normality of the distribution of the value data. Data normality test was performed using Shapiro Wilk. The distribution of the data is said to be normal if the *p* > 0.05, in this study the data was normally distributed with the *p* value = 0.118.

**Figure 3.**
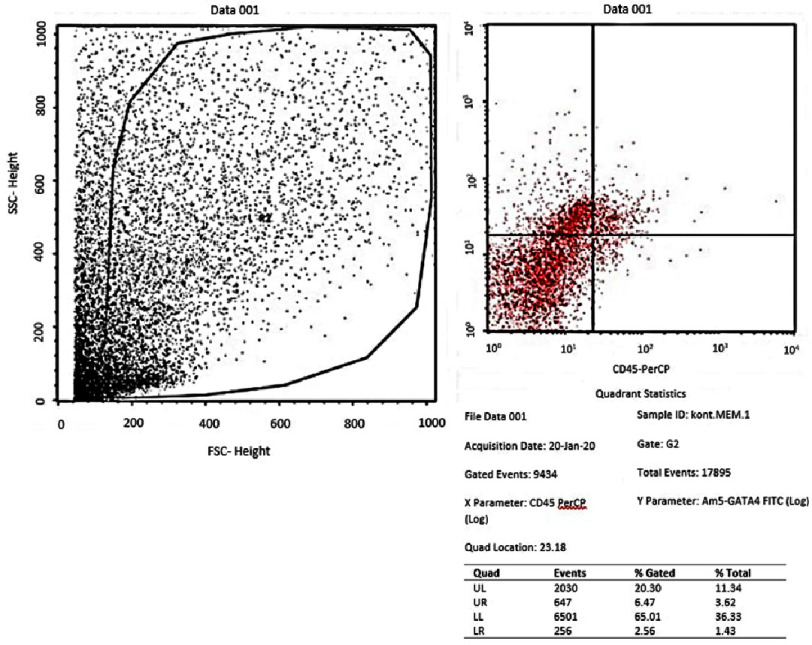
Flowcytometry of GATA-4 in the treatment group (medium of differentiation + PRF).

From the results of the flowcytometry examination of the three groups, the ratio data of GATA-4 (upper right quadrant) compared to the unexpressed GATA-4 (lower right quadrant) in the AMSCs group was an average of 58.1467 ± 1.23 in the negative control group (*α*-MEM) (Figure 1); 52.9633 ± 2.02 in the positive control group (medium of differentiation) (Figure 2); and 68.20 ± 6.82 in the treatment group (medium of differentiation + PRF) (Figure 3). This shows that the treatment group is superior to the negative control group and the positive control group is judged by the average ratio.

**Figure 4.**
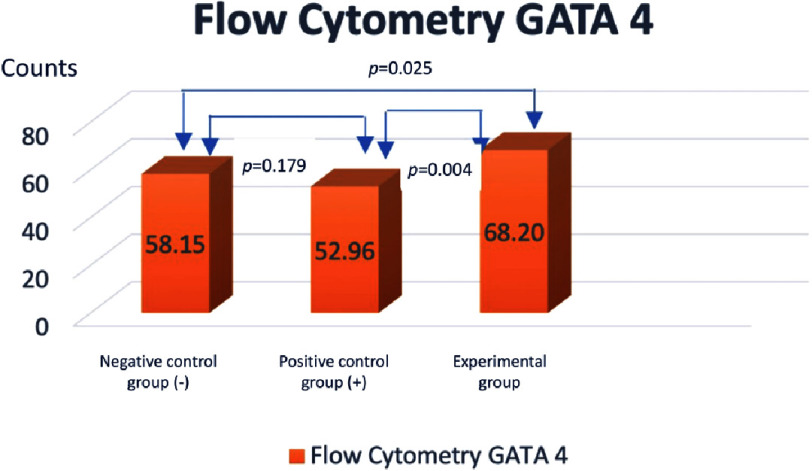
Bar diagram showing the ratio of GATA-4 expression divided to unexpressed GATA-4 in the three group of AMSCs.

**Figure 5.**
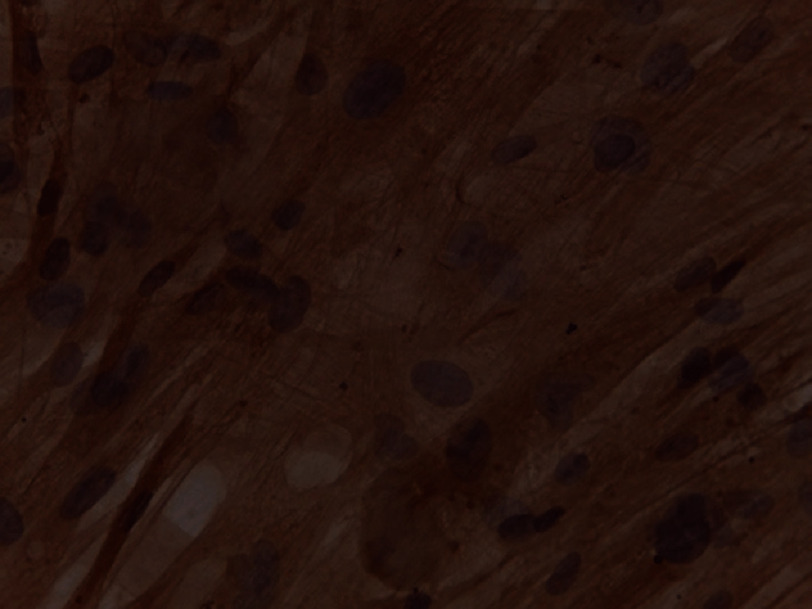
Immunocytochemistry of cTnT in the negative control group (*α*-MEM) with 400x magnification.

Because the distribution of the data is normal, ANOVA test was carried out to determine the difference between the three sample groups with *p* = 0.011. This showed a significant difference in the ratio of GATA-4 (upper right quadrant) compared to unexpressed GATA-4 (lower right quadrant) between the three groups. While the T-test was conducted to determine the difference between the two groups. There were significant differences between the negative control group (*α*-MEM) compared to the treatment group (differentiation medium + PRF) and the positive control group (differentiation medium) compared to the treatment group (differentiation medium + PRF) with *p* < 0.05. However, there was no significant difference between the negative control group (*α*-MEM) compared to the positive control group (medium of differentiation) with *p* = 0.179 (Figure 4).

**Figure 6.**
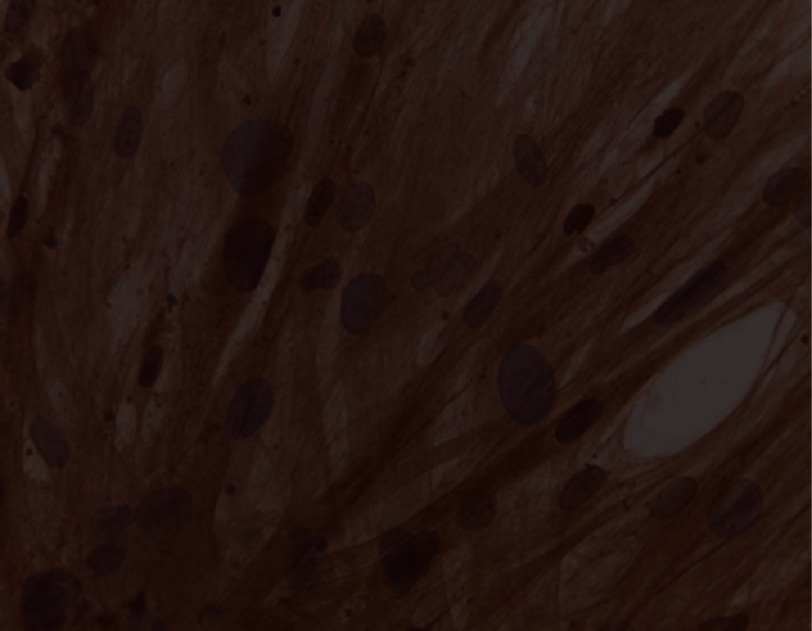
Immunocytochemistry of cTnT the positive control group (medium of differentiation) with 400x magnification.

### Immunocytochemical evaluation of troponin during cardiomyocyte differentiation

Cardiac Troponin T (cTnT), a late-stage marker of cardiomyocyte differentiation, becomes detectable from day 10. On day 10 of treatment, the three groups were assessed for immunocytochemical marker expression of cTnT. On immunocytochemistry examination, the determination of cardiomyocyte cells was morphologically visualized by staining the cytoplasm by DAB Chromogen and staining the nucleus by Meyer Hematoxylin.

Description of cardiomyocyte-like cells appear more prominent than other cells, with prominent blue nuclei and prominent brown cytoplasm. In the three groups, the accumulation of cardiomyocyte-like cells per visual field was calculated. To find out the difference test between the three groups, we began with a distribution test to determine the normality of the distribution of the value data. The Shapiro–Wilk test, selected due to the small sample size (n <30), confirmed normal distribution of the data (*p* = 0.315, normality criterion: *p* > 0.05).

**Figure 7.**
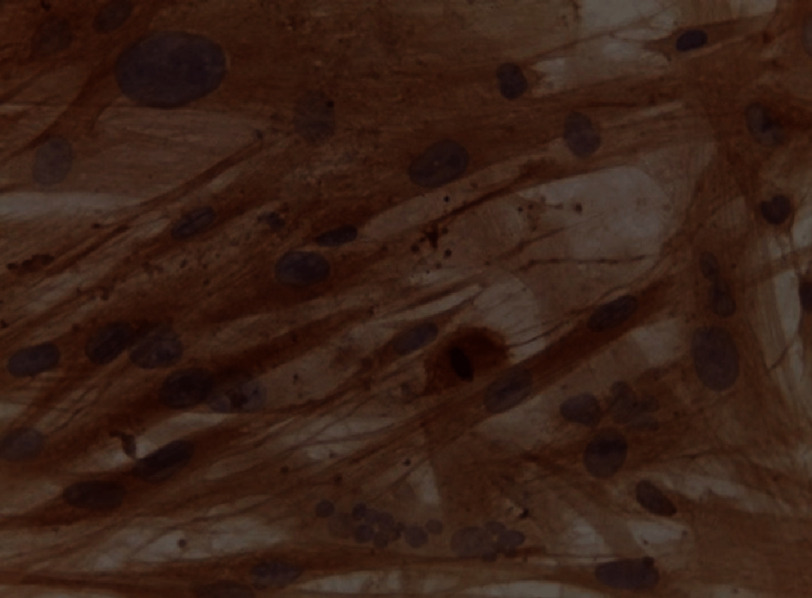
Immunocytochemistry of cTnT in the treatment group (medium of differentiation + PRF) with 400x magnification.

From the results of the immunocytochemistry examination of the three groups, the data obtained an average of 10.73 ± in the negative control group (*α*-MEM) (Figure 5); 26.00 ± 0.4 in the positive control group (medium of differentiation) (Figure 6); and 50.6 ± 7.2 in the treatment group (differentiation medium + PRF) (Figure 7). This shows that the treatment group is superior when compared to the negative control group and the positive control group based from the mean cTnT expression.

**Figure 8.**
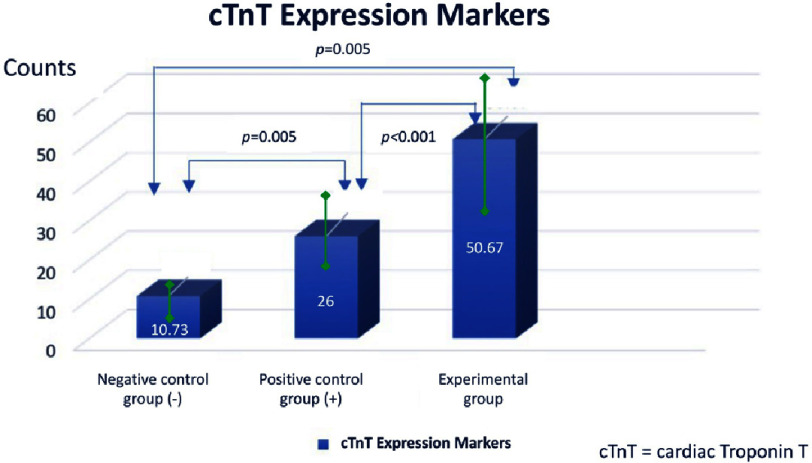
Bar diagram showing mean cTnT expression by immunocytochemistry in the three group of AMSCs.

Because the data were normally distributed, ANOVA test was carried out to determine the difference between the three sample groups with *p* = 0.0001. This shows that there is a significant difference in the mean of cardiomyocyte-like cells expressed with troponin between the three groups. While the T-test was conducted to determine the difference between the two groups. There were significant differences between the negative control group (*α*-MEM) compared to the treatment group (differentiation medium + PRF), the negative control group (*α*-MEM) compared to the positive control group (differentiation medium), and the positive control group (differentiation medium). compared with the treatment group (medium of differentiation + PRF) with *p* < 0.05 (Figure 8).

## Discussion

In this study, two examinations were carried out at the stage of differentiation of cardiomyocyte-like cells. In the first examination, namely at the cardiac progenitors stage in the treatment group that assessed GATA-4 expression by flow cytometry, it showed a significant increase in the PRF group compared to the negative control group and the positive control group (68.20 ± 6.82 *vs* 58.15 ± 1.23 *p* < 0.05; 68.20 ±6.82 *vs* 52.96 ± 2.02 *p* < 0.05). In the second examination, namely at the stage of mature cardiomyocyte-like cells assessing troponin expression with immunocytochemistry showed a significant increase in the PRF group compared to the negative control group and positive control group (50.66 ± 7.2 *vs* 10.73 ± 2.39 *p* < 0.05; 50.66 ± 7.2 *vs* 26.00 ± 0.4 *p*< 0.05). This is in accordance with the research hypothesis which states that there is an effect of adding PRF to the differentiation of AMSCs into cardiomyocyte-like cells.

Adipose tissue is a potential source of adult stem cells that can perform multipotent differentiation. Adipose-derived mesenchymal stem cells (AMSCs) have very similar properties to bone marrow-derived MSCs, including the ability to differentiate into osteoblasts, adipocytes and chondrocytes under certain *in vitro* conditions. Similar to other mesenchymal stem cells, various studies have shown that AMSCs have the potential to undergo adipogenesis, chondrogenesis, osteogenesis, myogenesis, and vasculogenesis. Because MSCs have many surface antigens, in 2006 the International Society of Cellular Therapy (ISCT) determined the minimum criteria for defining the culture of MSCs, namely (a) having plastic adherent properties on standard culture media, (b) positive expression of CD73, CD90, and CD105 , and negative expression of CD11b or CD14, CD19 or CD79a, CD45, and HLA-DR, and (c) have the potential for differentiation into adipocytes, chondrocytes, and osteoblasts using staining in *in vitro* cell cultures. This study used the expression phenotype characteristics of CD105+, CD34- and CD45- on AMSCs cultured in Passage-4 by flow cytometry technique. This is in accordance with the minimum criteria for the characterization of MSCs presented by ISCT^6^.

Coronary heart disease, among other cardiovascular diseases, has a very large influence on both length and quality of human life. Cardiomyocytes in an adult have a limited capacity to perform regeneration after coronary heart disease. Permanent damage to cardiomyocytes, loss of contraction function in the heart muscle and increased proliferation and turnover of fibroblast cells will lead to a progressive process of non-ischemic myocardial remodelling in the ventricles^7^. And this remodelling process will cause progressive ventricular dilatation and can result in heart failure. Currently there is no clinical therapy that has the effect of regenerating myocardium in coronary heart disease. Therefore, cell therapy is the most ideal therapy for regeneration of damaged myocardium^8^.

Platelet rich blood derivatives have been widely used in various fields of medicine and stem cells for tissue engineering because of their consistent ability to increase the potential for proliferation, migration and differentiation of stem cells. Platelet rich fibrin (PRF) is a new revolution in the concept of platelet therapy. Unlike platelet concentrates, this technique does not require a jellifying agent, but only centrifugation of natural blood without additives ^9,10,11,12^. Platelets are composed of granules including alpha granules, dense granules, and glycogen granules. Alpha granules are the main granules that contribute to wound healing through the various growth factors contained in it. Growth factors present in PRF include platelet derived growth factor (PDGF), transforming growth factor-*β*1 (TGF-*β*1), vascular endothelial growth factor (VEGF), insulin like growth factor-1 (IGF-1), epidermal growth factor (EGF), fibroblasts growth factor and others^11^.

The factor reaches the target cell and binds to transmembrane receptors and activates various intracytoplasmic proteins causing actions related to gene expression that have effects such as cell mitosis or collagen production. With the high content of growth factors possessed by PRF and the simplicity of manufacture, PRF can be an alternative for use in the field of cell therapy. Unfortunately, there are no studies linking the benefits of PRF with cardiomyocyte-like cells differentiation. This is what underlies this research. In previous studies, the optimum concentration of PRF varied from 50%, 10%, to less than 1%. Soffer et al., suggested a PRF of 0.5–1% as the optimum concentration for the rate of cell proliferation and mineralization^13^. However, Ferreira et al., found that 2% PRF was the optimum concentration for osteoblast proliferation^14^. In addition, Castegnaro et al. (2011)^15^, reported that 10% PRF was sufficient to induce cell proliferation in MSCs derived from adipose tissue^15^. In the study of Govindasamy et al. (2011) ^16^, 2% platelet lysate was referred to as the optimal concentration for mesenchymal stem cell and dental pulp stem cell proliferation and osteogenesis. Research conducted by Kang et al. (2011) stated that 2% PRF extract can increase the proliferation of human alveolar bone marrow stem cells (hABMSCs)^16^. In this study, the treatment group was given PRF with a concentration of 2%.

Similar to other mesenchymal stem cells, various studies have shown that AMSCs have the potential to undergo adipogenesis, chondrogenesis, osteogenesis, myogenesis, and vasculogenesis. Where are the various reagents from the previous study could induce AMSCs differentiation into cardiomyocyte-like cells including 5-azacytidine (5-Aza), angiotensin II (Ang II), and transforming growth factor-*β*1 (TGF-*β*1). In a study conducted by Safwani et al., in 2011 it was stated that 5-Aza was not effective for inducing cardio genesis in AMSCs. Therefore, in this study, it is suggested that the addition of growth factors is believed to help the induction of AMSCs. A study conducted by Planat Barnard et al., in 2004 confirmed that the use of growth factors for cardiomyocyte-like cells differentiation is necessary^17^.

The role of platelets in cardiomyocyte-like cells differentiation is said to lie in the growth factors contained therein. Where platelet activation that occurs in the cardiac recovery phase after ischemic damage causes the release of the alpha-granule component which is the main content of platelets which will mediate the formation of cardiac progenitor cells to become mature cardiomyocyte-like cells^18^. A limitation of the present study is that the blood samples and AMSCs were not examined *in vivo*, therefore we cannot study the immune incompatibility of the experimental results.

## Conclusion

In conclusion, we evaluated the beneficial effect of the addition of injectable platelet-rich fibrin on the differentiation of adipose-derived mesenchymal stem cells into cardiomyocyte-like cells in vitro. From the results above, increasing level of the expression of GATA-4 and Troponin means that PRF could accelerate differentiation of adipose-derived mesenchymal stem cells into cardiomyocyte-like cells; thereby contributing to the main processes of cardiac regeneration. However, further research is needed by examining more clinical, histological and statistical studies are required from different parts of the world to understand the benefits of platelet-rich fibrin at each stage of cardiomyocyte formation starting from cardiac mesoderm to mature cardiomyocyte-like cells.

## Data availability statement

The data that support the findings of this study are available from the corresponding author upon reasonable request.

## Acknowledgement

We would like to show our gratitude to Prof. Dr. Fedik Abdul Rantam as Consultant of Stem Cell at Tissue Bank, Dr. Soetomo Academic General Hospital and Prof. Dr. Maria Inge Lusida as Head of Institute of Tropical Disease, Universitas Airlangga for sharing their pearls of wisdom with us during the writing process, for their comments on the later version of the manuscript, although any errors are our own and should not tarnish the reputations of these esteemed persons. We would also like to thank for anonymous residents and staffs from Department of Cardiology and Vascular Medicine, Faculty of Medicine Universitas Airlangga - Dr. Soetomo Academic General Hospital for their technical contribution and so-called insights.

## Funding

No funding was received to perform or write this manuscript.

## Conflict of interest

The authors declare there are no competing interests.
